# Negative emotion recognition impairments despite preserved neural activity in patients with mild cognitive impairment (MCI)

**DOI:** 10.1162/IMAG.a.1428

**Published:** 2026-11-02

**Authors:** Rachana Mahadevan, Naomi Kristin Giesers, Thomas Liman, Karsten Witt, Andrea Hildebrandt, Mandy Roheger, Stephanie Rosemann

**Affiliations:** Ambulatory Assessment in Psychology Lab, Department of Psychology, Carl von Ossietzky Universität Oldenburg, Oldenburg, Germany; University Clinic of Neurology, Evangelical Hospital Oldenburg, Oldenburg, Germany; Charité – Universitätsmedizin Berlin, Center for Stroke Research Berlin, Berlin, Germany; German Center for Neurodegenerative Disease DZNE, Berlin, Germany; Research Center Neurosensory Science, University of Oldenburg, Oldenburg, Germany; Psychological Methods and Statistics, Department of Psychology, Carl von Ossietzky Universität Oldenburg, Oldenburg, Germany; Biological Psychology Lab, Department of Psychology, Carl von Ossietzky University Oldenburg, Oldenburg, Germany

**Keywords:** mild cognitive impairment, facial emotion recognition, socio-emotional functioning, neural activity, face processing

## Abstract

Emotion recognition is an essential component of socio-emotional functioning, yet its alteration in mild cognitive impairment (MCI) remains insufficiently understood, particularly in relation to neural mechanisms. This study examined behavioral and neural responses to facial emotions in 60 adults (30 MCI, 30 healthy controls (HC)) using a validated fMRI emotion-recognition paradigm with anger, fear, sadness, happiness, and neutral expressions. Although groups did not differ in overall recognition accuracy, a multivariate analysis revealed a significant group effect driven by a selective impairment in sadness recognition in MCI. Accuracy for anger and fear showed weaker, but non-significant differences between groups, and happiness recognition was preserved. Face-processing ability, emotional interference, emotional intelligence, and domain-specific cognitive performance showed no significant associations with facial emotion recognition, indicating that the sadness-specific deficit was not attributable to perceptual, cognitive, or socio-emotional characteristics. In fMRI analyses, HCs showed widespread activation to emotional versus neutral faces across occipital, frontal, and subcortical regions, whereas patients with MCI exhibited spatially more restricted activation. However, whole-brain group comparisons revealed no significant differences in BOLD response amplitudes for overall emotion processing or any emotion-specific contrast. These findings indicate that MCI is characterized by a selective impairment in recognizing sadness, an emotion relying on subtle, low-intensity cues, while whole-brain analyses did not reveal statistically significant group differences in neural responses during emotion processing. This pattern shows that group differences in behavior did not correspond to group differences in measured neural activity.

## 1 Introduction

Aging is a universal biological process that is associated with a progressive decline in cognitive function in some individuals. The severity of this decline, along with the subjective and cognitive complaints, helps determining the degree of neurodegeneration. When neurodegeneration is severe enough to manifest as dementia, a clinical syndrome characterized by decline across multiple cognitive domains, daily functioning becomes significantly impaired (Cipriani et al., 2020). In contrast, mild cognitive impairment (MCI), often considered a transitional stage between normal aging and dementia, involves a decline in one or more cognitive domains that does not interfere with daily activities and allows individuals to function independently (Gauthier et al., 2006). Notably, approximately 34% of patients with MCI progress to dementia over time, of whom 28% specifically develop Alzheimer’s disease. Meanwhile, around 45% remain stable and 15% revert to unimpaired cognition (Hu et al., 2017).

Cognitive decline in MCI is well established, the affected domains typically include memory, attention, executive functioning, language, and visuospatial abilities (Gauthier et al., 2006). These domains are not only central to general cognitive functioning but also contribute to emotional processing, including the perception, interpretation, and regulation of emotional information (Brosch et al., 2013). A recent meta-analysis (Roheger et al., 2022) has also identified deficits in emotion recognition, highlighting the wide range of socio-cognitive impairments associated with neurodegeneration. Recent investigations have focused on impairments in emotion recognition in dementia and its subtypes (González-Alcaide et al., 2021; Shi et al., 2025), particularly Alzheimer’s Disease. In contrast, a comparatively smaller number of studies (McCade et al., 2011; Morellini et al., 2022) have investigated this impairment in MCI, especially from the point of view of neural activation (Gandia-Ferrero et al., 2024; Park et al., 2017). Existing literature suggests that patients with MCI generally show deficits in the recognition of negative emotions (Elferink et al., 2015; McCade et al., 2011; Spoletini et al., 2008; Weiss et al., 2008), such as anger, fear, and sadness, while the recognition of happiness remains intact (Barbieri et al., 2022; Elferink et al., 2015). A recent large multimodal imaging study (Gandia-Ferrero et al., 2024) used the Reading the Mind in the Eyes Test (RMET) on 166 patients with MCI. They found that poorer emotion recognition performance correlated with lower gray matter volume in regions such as left straight gyrus, left superior frontal gyrus, right insula, anterior inferior cortex, right fusiform gyrus, right anterior cingulate gyrus, left anterior insular gyrus, and left anterior cingulate gyrus. Furthermore, emotion recognition was positively associated with standardized uptake values measured by fluorodeoxyglucose positron emission tomography (FDG-PET), reflecting regional glucose metabolism, such that lower metabolic activity in the medial prefrontal cortex, posterior cingulate, lateral temporal regions, insula, and basal ganglia was associated with poorer emotion recognition performance. These findings suggest that the functional and structural integrity of emotion-processing regions is linked to social cognitive performance in MCI and highlight the importance of combining behavioral and neuroimaging measures to capture the full extent of socio-emotional alterations. This informed our approach of examining both behavioral and neural responses to facial emotions using task-based fMRI, allowing us to investigate whether behavioral differences in emotion recognition are accompanied by alterations in neural activation patterns associated with emotion processing.

However, empirical evidence on alterations in neural activity during emotion recognition in patients with MCI is scarce. Understanding the emotional functioning of patients with MCI is crucial, as emotions play a key role in social interactions with our peers. Emotion recognition has been studied extensively across cultures, and while early theoretical frameworks proposed that certain facial expressions reflect biologically shared emotional signals (Ekman & Oster, 1979), this view has been increasingly challenged. Research suggests that the recognition of facial expressions is influenced by cultural context and that the concept of discrete basic emotions is itself contested (Jack et al., 2012; Ortony, 2022). Nevertheless, a set of frequently studied emotions, including anger, happiness, sadness, fear, disgust, and surprise, has been widely used in emotion recognition research and forms the basis of the present investigation. Emotion recognition is the ability to identify and interpret the emotional states of others, and it emerges early in life, for example, through mother–infant interaction, and is active from the first signs of cognitive processing or self-conscious awareness (Ekman & Oster, 1979). It involves directing attention to emotion-relevant sensory cues and then categorizing them in emotional terms (Spunt & Adolphs, 2019). This ability is crucial for maintaining social relationships, avoiding misunderstandings, and responding appropriately to potential threats. In MCI, deficits in emotion recognition can hinder effective communication and social interaction, potentially leading to frustration, conflict, or emotional withdrawal. These difficulties can also place an increased emotional strain on caregivers, contributing to caregiver burden (Pietschnig et al., 2016). Because caregiver burden reflects interpersonal strain arising from socio-emotional difficulties, it is relevant when examining individual variability in emotion-related functioning (Bucks & Radford, 2004). Higher burden is often reported when patients show more difficulty understanding or responding to others’ emotions, which can complicate daily interactions. Although caregiver burden is not a feature of MCI itself, it provides useful contextual information about the social environment surrounding the individual (Spitzer et al., 2019).

In addition to the cognitive changes associated with MCI, several individual factors have also been linked to differences in emotion recognition. For example, aging has been associated with reduced accuracy in identifying negative facial expressions (L. H. Phillips et al., 2002; Ruffman et al., 2008). This pattern is thought to reflect age-related decline in the recognition of specific negative emotions, particularly when assessed using static facial stimuli, with middle-aged adults performing at an intermediate level between young and older adults (Sze et al., 2012). Older adults also tend to show a positivity bias, with relatively preserved recognition of positive emotions alongside impaired recognition of negative ones (Di Domenico et al., 2015).

Cognitive functions further support emotion recognition by enabling the integration of perceptual information with stored emotional knowledge (Brosch et al., 2013). Attention directs processing toward emotionally relevant facial features and filters competing information (Oliveira et al., 2013; Schindler & Bublatzky, 2020), memory enables the matching of perceived expressions against categorical emotional representations formed through experience (Balconi & Fronda, 2021), and executive functioning enables the flexible integration of emotional cues with contextual information and the suppression of misleading features (Zimmerman et al., 2016). Importantly, these cognitive processes are also relevant specifically to MCI. In patients with amnestic MCI, poorer recognition of low-intensity fear has been linked to short-term verbal memory impairment (Spoletini et al., 2008), while facial emotion recognition has also been associated with attention and processing speed (Varjassyová et al., 2013). More recent evidence has further shown associations between facial emotion recognition deficits and executive and language functioning in MCI (Ju et al., 2024). Evidence from Parkinson’s disease-related MCI has also linked facial emotion recognition with visuospatial ability, alongside attention, executive function, and memory (Chiang et al., 2024).

MCI has also been associated with difficulties in inhibitory control, providing a rationale for examining whether emotional interference is related to facial emotion recognition performance (Sung et al., 2012). Furthermore, the ability to process faces holistically is also relevant, as recognizing emotional expressions depends on integrating individual facial features into a coherent representation rather than processing them in isolation (Richler & Gauthier, 2014). Patients with MCI have been shown to exhibit difficulties in face processing (Kawagoe et al., 2017; Lim et al., 2011), which may contribute to difficulties in recognizing emotional expressions.

Another factor relevant to interpersonal functioning is emotional intelligence (EI), which refers to the ability to perceive, understand, and manage emotions in oneself and others (Bru-Luna et al., 2021). Studies have shown that individuals with higher EI tend to show better accuracy when identifying emotional expressions, suggesting that EI may support the interpretation of affective cues and the regulation of emotional responses during social interaction (Gillioz et al., 2023; Hildebrandt et al., 2015). Because EI captures socio-emotional variability that is not explained by cognitive status alone, it is considered an important individual characteristic when studying emotion recognition (Agnoli et al., 2019).

Although direct evidence concerning trait EI specifically in relation to facial emotion recognition in MCI remains limited, EI has been linked to broader functioning in this population. In patients with MCI, EI was indirectly associated with well-being through cognitive function, instrumental activities of daily living, and social support (Saad et al., 2021). We, therefore, expected higher EI to be associated with better facial emotion recognition performance. A subsequent analysis of the same cohort using the emotion composite task, a different facial emotion recognition paradigm from the one used here, found that higher trait EI was associated with better performance (Mahadevan et al., 2026a), consistent with the expectation tested here. More broadly, the available literature provided an MCI-specific rationale for examining whether individual differences in cognitive functioning, emotional interference, face processing, and EI were associated with facial emotion recognition performance, and whether these associations differed between patients with MCI and HCs.

Studies in healthy individuals (Kim et al., 2015; Schirmer & Adolphs, 2017; Spunt & Adolphs, 2019) have identified several brain regions that are involved in emotion recognition, including the dorsomedial prefrontal cortex, ventrolateral prefrontal cortex, anterior temporal cortex, posterior superior temporal cortex, amygdala, fusiform gyrus, primary visual cortex, medial prefrontal cortex, precuneus, posterior cingulate cortex, thalamus, the somatosensory cortex (Kropf et al., 2019), and hypothalamus (Elferink et al., 2015; McCade et al., 2011). Specific emotions are associated with distinct neural correlates; for example, anger recognition has been associated with increased activation in the amygdala, fusiform face gyrus, superior temporal gyrus, and right inferior frontal gyrus (Sorella et al., 2021). Fear recognition has been associated with increased activation in the amygdala (Becker et al., 2012), pulvinar, and fronto-occipital regions (Di Tao et al., 2021). Sadness recognition has been associated with increased activation in the amygdala, right orbitofrontal and ventromedial prefrontal cortices, including the cingulate cortex (Arias et al., 2020; Nakajima et al., 2022). Happiness recognition has been linked to increased activation in the amygdala (Kipps et al., 2007), anterior insula (Chaminade & Spatola, 2022), medial prefrontal cortex (Nakajima et al., 2022), orbitofrontal cortices (Nakajima et al., 2022), and the hypothalamus (Chaminade & Spatola, 2022). Moreover, previous research provided evidence of reduced gray matter volume in brain regions such as the entorhinal cortex (located in the medial temporal lobe), hippocampus, and amygdala in patients with MCI (Csukly et al., 2016). Additionally, they exhibit reduced cortical thickness in regions such as the fusiform gyrus, amygdala, entorhinal cortex, precuneus, and cingulate cortex compared with HCs.

Classic theories emphasize that emotions are a set of discrete and universal categories (Ekman & Oster, 1979); however, more recent theoretical frameworks have challenged this view. For example, a recent meta-analysis (Lindquist et al., 2012) argues that emotional experiences arise from distributed, domain-general processes such as conceptualization, attention, and memory. Rather than mapping one-to-one onto specific regions, emotional states emerge from coordinated activity across large-scale networks, including default-mode, salience, and frontoparietal control systems.

Although emotional processes may rely on distributed brain networks, certain regions have been consistently linked to specific aspects of emotion perception. The amygdala is involved in detecting emotionally salient stimuli (Becker et al., 2012), the fusiform gyrus supports face perception and the integration of facial features (Richler & Gauthier, 2014), and the insula contributes to interoceptive and affective awareness (Kropf et al., 2019). These regions operate within broader large-scale systems implicated in emotion processing, including salience and default-mode networks that support emotional evaluation, internally directed processing, and social cognition (Lindquist et al., 2012). Examining activity in these regions, therefore, remains informative, particularly in clinical groups such as MCI, where broader network-level alterations may manifest through changes in activation within key emotion-processing areas. For this purpose, we conducted a functional magnetic resonance imaging (fMRI) study with patients with MCI and healthy controls (HCs). We used a facial emotion recognition task with stimuli from the Radboud Face Database (Langner et al., 2010), which included emotions such as anger, fear, sadness, happiness, and neutral. While emotional processing likely reflects distributed, domain-general networks, prior empirical work consistently identifies contributions from specific regions to particular emotional processes. Based on this literature, we formulated the following three hypotheses: H1a: We expected patients with MCI to perform worse in facial emotion recognition compared with HCs. Consistent with the literature, we expected this impairment to be more pronounced for negative emotions such as anger, fear, and sadness, while recognition of happiness was expected to remain relatively intact (Barbieri et al., 2022; Elferink et al., 2015). H1b: We further expected that better performance on cognitive measures, including memory, attention, executive functioning, language, and visuospatial abilities, as well as higher emotional intelligence, better face processing performance, and lower emotional interference, would be associated with better facial emotion recognition performance (McCade et al., 2011; Spoletini et al., 2008; Weiss et al., 2008). We additionally examined whether these associations differed between patients with MCI and HCs. H2: We expected patients with MCI to show lower BOLD response amplitudes compared with HCs in core emotion-processing regions including the fusiform gyrus, amygdala, posterior superior temporal sulcus, anterior temporal cortex, insula, secondary somatosensory cortex, ventrolateral prefrontal cortex, and dorsomedial prefrontal cortex, when processing emotional faces relative to neutral faces (Kim et al., 2015; Schirmer & Adolphs, 2017; Spunt & Adolphs, 2019). H3: In terms of individual emotions, we expected lower BOLD response amplitudes for negative emotions such as anger (amygdala, fusiform gyrus, superior temporal gyrus, right inferior frontal gyrus (Sorella et al., 2021)), fear (amygdala, pulvinar, frontal-occipital regions (Di Tao et al., 2021)), and sadness (right orbitofrontal and ventromedial prefrontal, including cingulate cortex (Nakajima et al., 2022)) in patients with MCI in comparison with the HCs (Barbieri et al., 2022; Elferink et al., 2015).

## 2 Methods

Our study was pre-registered prior to data collection at the Open Science Framework (https://osf.io/yn3gp). We followed the TREND Statement Checklist (Des Jarlais et al., 2004) for reporting (refer to Supplementary Table S1). The results of more detailed behavioral testing are reported in Mahadevan et al. (2026a).

### 2.1 Participants

The study was planned and conducted at the Department of Psychology, Carl von Ossietzky Universität of Oldenburg, Germany, from October 2023 to December 2024. The study included a total of 60 participants with an age range of 50 to 86 years (*M* = 66.8, *SD* = 8.7), and the participants were further classified as patients with MCI (*n* = 30; age: *M* = 70.5, *SD* = 8.2; 14 women, 16 men, 0 diverse) and HCs (*n* = 30; age: *M* = 63.1, *SD* = 7.6; 17 women, 13 men, 0 diverse). Participants self-reported their gender during screening, and this information was recorded using the categories woman, man, or diverse. The patients with MCI were recruited from the Evangelisches Krankenhaus Oldenburg, and the HCs were recruited via newspaper advertisements. Classification of MCI was based on cognitive screening using the Montreal Cognitive Assessment (MoCA) (Nasreddine et al., 2005). Individuals scoring between 18 and 25 were considered to show cognitive impairment consistent with MCI, while scores ≥26 indicated healthy performance and were used to classify HCs. Group allocation in the present study was based on predefined study criteria using MoCA screening scores. To be included in the study, participants had to be 50 years of age or older and below 90 years of age, fluent in German, without a history of substance dependence in the past 6 months, and without any diagnosed neurological or psychiatric disorders. Furthermore, during the screening session, it was ensured that participants could function independently as measured by the Instrumental Activities of Daily Living (Lawton & Brody, 1969) (IADL; with a cutoff of 6 points or more) and that they did not have any underlying anxiety or depression, as measured by the Hospital Anxiety and Depression Scale (Zigmond & Snaith, 1983) (HADS; with a cutoff of <8 for HCs and <11 for patients with MCI. A stricter threshold was applied to HCs to exclude even borderline mood symptoms, whereas the threshold for patients with MCI was based on the established clinical cutoff in order to avoid excluding individuals with mild subclinical mood symptoms that are relatively common in this population (Ismail et al., 2017). Since we collected MRI data, we filtered out participants who had implants that were not suitable for the MRI. Of the 79 individuals who were initially recruited, 9 were excluded due to MRI contraindications and 3 withdrew due to personal commitments, leaving 67 who were invited to the laboratory session. Of these, two were excluded for high HADS scores and one for a MoCA score below 18. Following screening, 34 participants were classified as HC and 30 as patients with MCI. At the MRI session, 2 HC participants were excluded due to technical errors in data collection and 2 did not complete the measurement, resulting in a final sample of 60 participants (30 HC, 30 MCI). All 60 were included in the fMRI and primary behavioral analyses. Two participants were excluded from analyses involving the facial composite task due to technical errors, and two were excluded from the Emotional Stroop analyses due to color blindness. A flowchart containing a detailed summary of the flow of participation is also reported in Mahadevan et al. (2026a).

### 2.2 Procedure

The study was carried out in a structured three-phase process, where at first, the interested participants were contacted via a telephone interview, which assessed the basic eligibility criteria only. Following this, those who were eligible to take part in the study were invited to the first testing session in the laboratory, where they had to sign a written consent form confirming that they were clear of the study procedure. This session further included all standardized assessments, including the MoCA, IADL, and HADS, a series of neuropsychological assessments via the CERAD-NAB, the Trait Emotional Intelligence Questionnaire – Short Form (TEIQUE-SF) (Chirumbolo et al., 2019; Petrides, 2009), and computer tests that measured general face processing via the facial composite task (Petrakova et al., 2018), emotion processing via the facial emotion composite task (Wilhelm et al., 2014), and emotional interference via the Emotional Stroop Task (Ben-Haim et al., 2016), respectively. The second session was the MRI measurement at the Neuroimaging unit of the Carl von Ossietzky Universität of Oldenburg.

### 2.3 Facial emotion recognition task

To measure the facial emotion recognition abilities and their related BOLD response amplitudes, we designed a facial emotion recognition task that consisted of static images from the Radboud faces database (RaFD; Langner et al., 2010). The RaFD has also been implemented in previous studies of facial emotion recognition involving older adults and older clinical populations (Cousins et al., 2021; Visser, 2020). The task was administered using the Presentation® software (version 23.0, Neurobehavioral Systems, Inc., Berkeley, CA, www.neurobs.com), and the participants performed this task in the MRI. This task started with a trial round outside the MRI to ensure the participants were familiar with the response buttons. The main task consisted of 150 trials with 30 trials for each emotion (anger, sadness, fear, happiness, and neutral). We included these four basic emotions because of their association with three core affects, namely, reward (happiness), punishment (sadness), and stress (fear and anger) (Gu, Wang, Cao, et al., 2019; Gu, Wang, Patel, et al., 2019). These emotions represent only the typical affect core, that is, either valence or arousal. While also a basic emotion, disgust primarily reflects a strong arousal response, manifesting as intense aversion or dislike ranging from unpleasant odors to injustice and immoral behavior. In contrast, surprise often represents a reaction to stimuli rather than a distinct emotional state, and its valence depends heavily on context. For example, unexpected news could be a promotion or the death of a loved one (Gu, Wang, Cao, et al., 2019; Gu, Wang, Patel, et al., 2019). Therefore, we excluded disgust and surprise because they do not clearly align with the targeted core emotional dimensions. Furthermore, we chose neutral as the baseline emotion because previous studies have shown that, when visual and semantic factors are matched, neutral stimuli provide a stable reference point. This allows us to identify emotion-related increases in neural similarity and arousal effects (Riberto et al., 2022) and represents an ecologically valid anchor, given that emotional expressions typically arise from a neutral facial state (Vikhanova et al., 2022).

In the task, each trial began with a fixation cross (250 ms) appearing at the center of the screen, followed by the stimulus display for 2700 ms. After this, four response options appeared, which were randomly selected (it was always the correct emotion and three other possible emotions). The response window was 5000 ms, and the participants could answer via an fMRI-compatible response pad (LXPAD-2 x 5–10M, NAtA technologies, Coquitlam, Canada) using the middle and index finger from both hands (see also Fig. 1). To select their response, participants pressed one of four buttons on the response pad. The four emotion labels changed position on every trial, so participants could not rely on fixed button emotion associations. Thus, in total, each trial lasted for 8 seconds, and the total duration of the task was 20 minutes. The visual stimuli were presented by a projector (DLA-G15E, JVC Professional, Japan) mounted on the rear end of the scanner, and 50 cm was maintained from the eye to the screen.

**Fig. 1.**
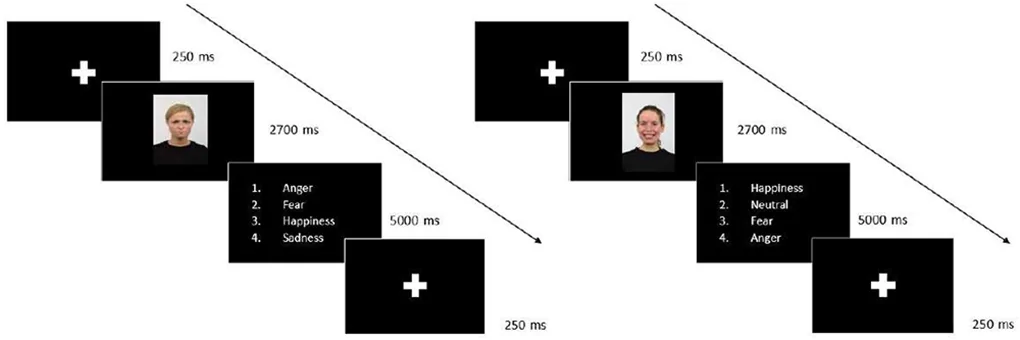
Overview of the facial emotion recognition task performed in the MRI scanner.

### 2.4 fMRI data acquisition

The MRI data were acquired via a 3T whole-body Siemens Magnetom Prisma MRI with a 20-channel head coil. Functional data were recorded while the participants performed the facial emotion recognition task in the scanner. T2*-weighted images were acquired with a gradient echo planar imaging (EPI) sequence (1424 volumes, TR = 850 ms, TE = 30 ms, voxel size = 2.5 × 2.5 × 2.5 mm, flip angle = 62 degrees, 48 slices). This was followed by the acquisition of the T1-weighted structural images (MP-RAGE, TR = 2000 ms, TE = 2.07 ms, voxel size = 0.8 × 0.8 × 0.8 mm, flip angle 9 degrees, 224 sagittal slices). During the data acquisition, there was a software update of the MRI from VE11C to XA30. The sequence parameters themselves did not change after the update, which we confirmed with a repeated pilot scan. Following this point, we acquired data from 18 participants (MCI = 11, HC = 7).

### 2.5 Additional behavioral testing

Participants underwent a broader behavioral assessment in addition to the facial emotion recognition task. Global cognitive function was measured using the MoCA (Nasreddine et al., 2005). Scores ranging from 18 to 25 were classified as MCI, while scores between 26 and above indicated normal performance and were classified as HCs. Domain-specific abilities in memory, attention, executive functioning, language, and visuospatial construction were assessed using the Consortium to Establish a Registry for Alzheimer’s Disease Neuropsychology Battery (CERAD-NAB) (Morris et al., 1989). Group classification was based on MoCA performance rather than CERAD-NAB scores. The CERAD-NAB domain scores were subsequently used as continuous variables in the behavioral analyses. EI was assessed using the Trait Emotional Intelligence Questionnaire – Short Form (TEIQUE-SF) (Petrides, 2009), comprising 30 items based on 4 sub-scales, including well-being, self-control, emotionality, and sociability. Face processing abilities were measured via the facial composite task (Petrakova et al., 2018), where the participants had to judge the similarity of facial halves across 40 trials, that is, participants had to judge whether the cued half of 2 faces that were shown one after the other were the same. Finally, emotional interference was evaluated using a computerized Emotional Stroop Task (Ben-Haim et al., 2016). Participants named the ink color of neutral and emotionally valanced words, and the emotional Stroop effect was calculated as the reaction-time difference between emotional and neutral trials, providing an index of interference from emotional content. This effect reflects attentional competition, where emotional content involuntarily captures attention and slows task-relevant processing. Together, these measures allow us to examine the perceptual, cognitive, and socio-emotional factors that contribute to facial emotion recognition. This provides a deeper perspective on how difficulties in processing facial emotions may arise in MCI. Perceptual tasks such as the facial composite task and the emotional Stroop task capture the lower-level visual and attentional demands involved in processing facial and emotional cues. Meanwhile, domain-specific neuropsychological measures index memory, language, and executive functions that support the integration and interpretation of emotional information. Socio-emotional measures (like EI) reflect interpersonal and emotional context. This multi-level approach enables a more comprehensive understanding of the behavioral and neural correlates of emotion recognition in MCI.

### 2.6 Analysis

#### 2.6.1 Analysis of behavioral data

The behavioral analysis was conducted using R (v4.1.2; R Core Team, 2021). We first computed descriptive statistics for all continuous variables, including demographic measures (age, education), EI scores, and neurocognitive scores from the CERAD battery and the MoCA. Group differences between the HCs and patients with MCI were initially examined using independent *t*-tests to provide an overview of baseline behavioral differences. To formally test our primary hypothesis (H1a) regarding facial emotion recognition performance, we implemented a series of general linear models and multivariate general linear models. As a first step, we estimated a general linear model assessing whether overall facial emotion recognition accuracy differed between groups when adjusting for age. To evaluate whether these group differences extended to specific emotional categories, we conducted a multivariate analysis of variance (MANOVA) with anger, fear, happiness, neutral, and sadness accuracy entered as simultaneous dependent variables and group and age entered as predictors. To further investigate mechanistic contributions to facial emotion recognition, two additional MANOVAs tested whether general face processing ability (as measured by the facial composite task) and emotional interference (emotion Stroop effect reaction times) moderate the association between group status and emotional accuracy. These models included Group × facial composite task scores and Group × emotion Stroop effect reaction times as interaction terms to evaluate moderation effects while adjusting for age. Following the multivariate analyses, we estimated a series of domain-specific general linear models that examined the relationship between facial emotion recognition performance and EI, demographic characteristics (age, gender, education), and a comprehensive set of cognitive measures such as memory, attention, language, visuospatial construction, and executive functioning (H1b). Each domain model included Group × predictor interactions to determine whether the association between cognitive performance and facial emotion recognition differed between HC and MCI. The raw *p*-values were corrected for multiple comparisons using the false discovery rate (FDR) where necessary to ensure appropriate control of Type I error given the number of statistical tests conducted. All behavioral models were checked for key statistical assumptions. For the univariate models, we tested residual normality (Shapiro–Wilk), homoscedasticity (Breusch–Pagan), and multicollinearity (VIF). Residuals showed some non-normality, which is typical for bounded accuracy data, but there was no sign of model misspecification; homoscedasticity was met, and VIF values were acceptable except for the expected increases in models with interactions. For the MANOVAs, we assessed assumptions using Mardia’s tests, Box’s M test, and Mahalanobis distance. Multivariate normality was not met, but covariance matrices were homogeneous and only one mild outlier was found, indicating that the MANOVA results remain robust.

#### 2.6.2 Analysis of fMRI data

The analysis of the fMRI data was conducted in SPM12 based on Matlab (version 2023a). The analysis pipeline included the following stages: (i) *Preprocessing in the following order:* spatial realignment estimation, slice-time correction, co-registration, segmentation, normalization to the Montreal Neurological Institute (MNI) space using parameters obtained from the segmentation of anatomical T1-weighted image, and smoothing (full-width-at-half-maximum = 8 mm), (ii) in the first level of analysis, a temporal high-pass filter (128 seconds) was applied and the temporal autocorrelations across the scans were modeled using an AR (1) model. We modeled four different conditions (sadness vs neutral, anger vs neutral, fear vs neutral, happiness vs neutral) individually with the canonical hemodynamic response function. Each trial was modeled using the stimulus display window (2.7 seconds), beginning at stimulus onset. The response period was not included in the model. Regression coefficients (betas β) for each regressor at each measured voxel in the brain and linear contrasts between these betas were calculated using the general linear model. Head motion was quantified using framewise displacement (FD) derived from SPM realignment parameters. Mean FD differed slightly between groups (HC: *M* = 0.2177 mm, *SD* = 0.0802; MCI: *M* = 0.2662 mm, *SD* = 0.0765; *t* = –2.40, *p* = 0.020); however, overall motion was low in both groups (<0.3 mm). Motion covariates (six realignment parameters) were included in the first-level analysis to control for residual motion effects, (iii) *second-level analysis* was performed once we obtained the β maps from the first-level analysis. The goal of the second-level analysis was to compare group BOLD response amplitudes. At first, a model was built using a one-sample *t*-test to assess the BOLD response amplitudes within the groups (HC and MCI separately), and the second step was to do a between-group comparison of BOLD response amplitudes using a two-sample *t*-test (HC vs MCI, i.e., HC > MCI and MCI > HC). The second-level analyses were computed on the whole-brain level. For the fMRI data, effects were determined to be significant when passing a threshold of *p* < 0.05 (FWE cluster size inference with *p* = 0.001 cluster-forming threshold). Age was added as a covariate to account for age-related variance between groups. Peak coordinates are reported in MNI space.

## 3 Results

### 3.1 Demographic and cognitive characteristics

The results of the *t*-test comparisons revealed that the groups differed significantly in age (HC: *M* = 63.1, *SD* = 7.6; MCI: *M* = 70.5, *SD* = 8.1; *t*(58) = -3.64, *p* < 0.01, *d* = 0.94), with patients with MCI being older than HCs. In terms of cognitive performance, MoCA scores were significantly lower in patients with MCI (HC: *M* = 27.7, *SD* = 1.3; MCI: *M* = 23.1, *SD* = 1.6; *t*(58) = 12.57, *p* < 0.01, *d* = 3.25), confirming the presence of cognitive decline in this subsample. Patients with MCI also scored significantly lower on the facial composite task (HC: *M* = 27.8, *SD* = 4.2; MCI: *M* = 23.6, *SD* = 5.4; *t*(56) = 3.22, *p* < 0.05, *d* = 0.85), indicating reduced face processing ability. Group differences were additionally observed in EI (HC: *M* = 5.74, *SD* = 0.58; MCI: *M* = 5.27, *SD* = 0.74; *t*(58) = 5.74, *p* < 0.01, *d* = 0.71), and in immediate memory (*t*(58) = 5.56, *p* < 0.01, *d* = 1.44), confirming broader cognitive and socio-emotional differences between groups. A detailed summary of all descriptive statistics including range values and effect sizes is provided in Supplementary Table S2.

### 3.2 Behavioral results

To test H1a, we conducted a general linear model and MANOVA examining whether patients with MCI differed from HCs in facial emotion recognition overall and across specific emotion categories. Overall, patients with MCI showed numerically lower recognition accuracy across all facial emotions compared with HCs. The difference was statistically significant only for sadness (HC: *M* = 84.7, *SD* = 19.8; MCI: *M* = 66.2, *SD* = 26.1), while differences for anger (HC: *M* = 82.7, *SD* = 14.9; MCI: *M* = 74.0, *SD* = 20.2), fear (HC: *M* = 90.8, *SD* = 14.7; MCI: *M* = 82.1, *SD* = 23.6), happiness (HC: *M* = 94.0, *SD* = 13.3; MCI: *M* = 88.8, *SD* = 20.0), and neutral expressions (HC: *M* = 84.9, *SD* = 15.4; MCI: *M* = 83.8, *SD* = 19.9) did not reach statistical significance, suggesting a selective rather than global deficit pattern. These results are summarized in Table 1. Figure 2 visualizes the distribution of total ERT performance across groups. To assess overall group differences in total recognition performance, we first estimated a general linear model predicting total facial emotion recognition accuracy. Group did not significantly predict overall performance (β = −0.33, *p* = 0.242, FDR *p* = 0.242), and age was also not associated with accuracy (β = −0.19, *p* = 0.176, FDR *p* = 0.242), indicating that overall recognition ability did not differ between groups (see Supplementary Table S3). We then examined whether group differences varied across specific emotions using a MANOVA including anger, fear, happiness, neutral, and sadness accuracy as dependent variables, with group and age as predictors. The multivariate group effect was significant, Wilks’ λ = 0.790, *F*(5,53) = 2.83, *p* < 0.05, indicating that MCI and HC participants showed different emotion-specific performance profiles. Age was not significant (*p* = 0.370). Follow-up univariate ANOVAs showed that this multivariate effect was mainly driven by lower sadness recognition in MCI (*p* < 0.05), while the effects for anger (*p* = 0.062) and fear (*p* = 0.090) were smaller and they were not significant and hence did not support reliable group differences. No group differences were found for happiness (*p* = 0.241) or neutral expressions (*p* = 0.810). These univariate tests were to clarify which emotions contributed most to the multivariate pattern. A detailed summary of these results is given in Supplementary Table S4.

**Fig. 2.**
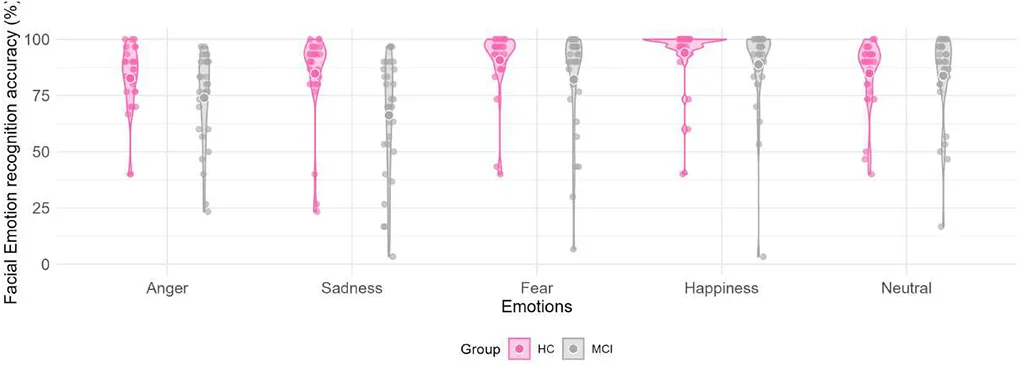
A violin plot showing emotion-specific performance on the facial emotion recognition task across groups (HC and MCI).

**Table 1.** Facial emotion recognition performance (% correct) by group.

	HC		MCI	
Emotions	Mean percentage	SD	Mean percentage	SD
Anger	82.7	14.9	74	20.2
Sadness	84.7	19.8	66.2	26.1
Fear	90.8	14.7	82.1	23.6
Happiness	94	13.3	88.8	20
Neutral	84.9	15.4	83.8	19.9

*Note.* Values indicate the mean percentage and standard deviation (SD) of correct responses for each emotion category. Each category comprised 30 trials (150 total). Data reflect raw, unadjusted accuracy scores before any model-based correction.

Next, we tested whether facial processing ability (as measured by the facial composite task) explained group differences. A MANOVA including group, facial composite task scores, and age showed a significant multivariate group effect, Wilks’ λ = 0.755, *F*(5,49) = 3.18, *p* < 0.05, but the facial composite task scores did not predict multivariate performance (Wilks’ λ = 0.921, *p* = 0.528), and the interaction with the group was not significant (Wilks’ λ = 0.911, *p* = 0.452). As in the previous model, sadness showed the clearest group difference, but facial composite task performance did not explain accuracy for any emotion. This indicates that reduced face perception ability did not account for the emotion-specific impairment in MCI. A detailed summary of these results is given in Supplementary Table S5. We also examined whether emotional interference (ESE reaction times) explained group differences. The multivariate group effect remained significant (Wilks’ λ = 0.773, *F*(5,49) = 2.88, *p* < 0.05), whereas neither emotional interference (ESE reaction times; Wilks’ λ = 0.990, *p* = 0.991) nor its interaction with the group (Wilks’ λ = 0.971, *p* = 0.917) contributed to the model. Again, sadness showed the strongest univariate group difference, while effects for other emotions were weaker. Emotional interference, therefore, did not explain the selective impairment observed in MCI. A detailed summary of these results is given in Supplementary Table S6. To test H1b, we estimated a series of general linear models examining whether cognitive performance, EI, face processing ability, and emotional interference were associated with facial emotion recognition performance and whether these associations differed between groups. Across models, no group × predictor interactions remained significant after FDR correction. Although several predictors showed associations at the raw-p level (e.g., immediate memory, visuospatial recall, word recognition), none survived correction (see Supplementary Tables S7–S13). Thus, the selective impairment in sadness recognition in MCI was not attributable to general cognition, emotional intelligence, or demographic variables. Taken together, these results show that patients with MCI exhibit a distinct emotion-specific deficit, most clearly in the recognition of sadness, while overall emotion recognition ability remains comparable with that of HCs. Figure 2 shows the distribution of total ERT performance across groups.

### 3.3 FMRI results

#### 3.3.1 Overall facial emotion recognition

To test H2 and H3, we conducted whole-brain second-level analyses in SPM12 comparing BOLD response amplitudes between groups for the overall emotional versus neutral contrast and for each individual emotion contrast separately. In the HCs, this contrast yielded increased BOLD response amplitudes across the left occipital pole, left middle temporal gyrus, right caudate, left triangular part of the inferior frontal gyrus, cerebral vermis lobules, and the posterior cingulate gyrus. Whereas, in patients with MCI, increased BOLD response amplitudes were more restricted and observed in limited regions, primarily in the left occipital pole and the left triangular part of the inferior frontal gyrus. Of the regions hypothesized in H2, activation in the inferior frontal gyrus was observed in HCs during overall emotion processing relative to neutral faces. However, the amygdala, insula, posterior superior temporal sulcus, anterior temporal cortex, secondary somatosensory cortex, and dorsomedial prefrontal cortex did not reach significance in either group.

Figure 3 shows the activation patterns for overall emotion recognition based on the contrast of all emotional faces vs. neutral faces (emotional > neutral), for each group separately: left—HCs and right—patients with MCI. Table 2 displays the peak MNI coordinates for overall emotion recognition relative to neutral for each group.

**Fig. 3.**
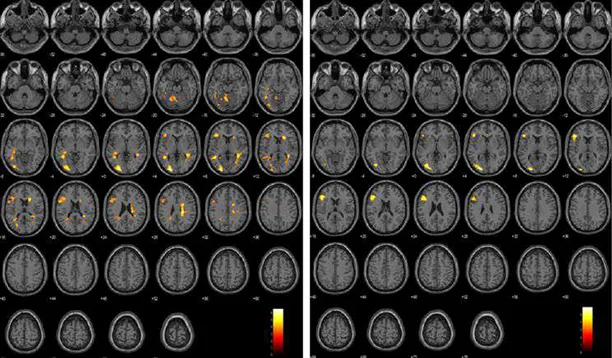
The activation patterns for the overall emotion recognition based on the contrast of all emotional faces vs. neutral faces (emotional > neutral), for each group, are shown separately: left—HCs and right—patients with MCI [*p* < 0.05; FWE corrected at the cluster level].

**Table 2.** Peak MNI coordinates for overall emotion recognition vs neutral for each group.

Group	Peak coordinate (x, y, z)	*Z*-value	Cluster size (k)	Brain region
HC	(-24, -94, 6)	5.08	857	Left occipital pole
	(-42, -48, 6)	4.55	861	Middle temporal gyrus
	(22, 20, 12)	4.43	251	Right caudate
	(-40, 30, 8)	4.16	796	Left triangular part of the inferior frontal gyrus
	(-8, -54, -18)	4.05	381	Cerebral vermis lobules I–IV
	(-22, -48, 28)	3.83	262	Posterior cingulate gyrus
MCI	(-22, -92, 6)	4.76	458	Left occipital pole
	(-44, 26, 12)	4.28	803	Left triangular part of the inferior frontal gyrus

Next, we compared neural activity between the two groups (HC > MCI and MCI > HC). This comparison did not reveal any significant differences in BOLD response amplitudes between the two groups.

#### 3.3.2 Emotion-specific results

Further, we also examined the bold response amplitudes to each of the four individual emotions separately within each group, relative to the neutral condition. Table 3 displays the peak MNI coordinates for different stimulation conditions for each group.

**Table 3.** Peak MNI coordinates for different stimulation conditions in the facial emotion recognition task for each group.

Condition	Group	Peak coordinate (x, y, z)	Z-value	Cluster size (k)	Brain region
Anger	HC	(-56, 14, 4)	5.78	2418	Left opercular part of the inferior frontal gyrus
		(-44, -48, -4)	5.47	2630	Left fusiform gyrus
		(38, -64, -20)	4.91	1239	Right occipital fusiform gyrus
		(44, -22, 62)	4.78	1908	Right postcentral gyrus
		(-34, -18, 26)	4.44	386	Left central operculum
		(-4, 0, 60)	4.37	381	Left supplementary motor cortex
Sadness	HC	(-26, -92, 2)	5.11	5168	Left inferior occipital gyrus
		(22, -26, 28)	5.08	468	Right anterior parahippocampal gyrus
		(-38, 32, 6)	5.07	3303	Left triangular part of the inferior frontal gyrus
		(38, -46, 12)	4.76	302	Right middle temporal gyrus
		(-26, -50, 10)	4.25	279	Left precuneus
		(-36, -26, 64)	4.06	494	Left postcentral gyrus
		(18, 12, 18)	4.01	307	Right caudate
		(-4, 6, 60)	4.00	289	Left supplementary motor cortex
Fear	HC	(-24, -92, 6)	6.50	7455	Left inferior occipital gyrus
		(-40, 30, 10)	5.15	3422	Left triangular part of the inferior frontal gyrus
		(22, -26, 28)	4.36	472	Right anterior parahippocampal gyrus
		(20, 22, 14)	4.12	324	Right caudate
Happiness	HC	(-6, 26, -4)	4.82	1488	Left anterior cingulate gyrus
		(-50, 6, -26)	4.13	286	Left temporal pole
Anger	MCI	(-48, 28, 10)	5.20	1814	Left triangular part of the inferior frontal gyrus
		(-26, -88, 6)	4.56	432	Left Inferior occipital gyrus
Sadness	MCI	(48, -38, 6)	5.37	3193	Right middle temporal gyrus
		(30, -88, 8)	5.23	477	Right inferior occipital gyrus
		(-40, 24, 22)	4.99	2292	Left middle frontal gyrus
		(-28, -52, 36)	4.37	387	Left superior parietal lobule
Fear	MCI	(-24, -92, 6)	5.10	1092	Left inferior occipital gyrus
		(-40, 26, 12)	4.94	1592	Left triangular part of the inferior frontal gyrus

**Anger** was associated with increased BOLD response amplitudes in the left opercular part of the inferior frontal gyrus, the right and left fusiform gyrus, the right postcentral gyrus, left central operculum, the left supplementary motor cortex in the HCs. For MCI, anger activated the left triangular part of the inferior frontal gyrus and the left inferior occipital gyrus. Figure 4 shows the activation patterns for the anger condition, based on the contrast of anger faces vs. neutral faces (anger > neutral), for each group separately: left—HCs and right—patients with MCI.

**Fig. 4.**
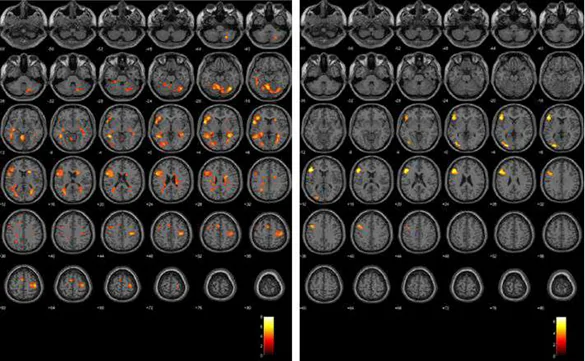
The activation patterns for the anger condition, based on the contrast of anger faces vs. neutral faces (anger > neutral), for each group, are shown separately: left—HCs and right—patients with MCI [*p* < 0.05; FWE corrected at the cluster level].

**Sadness** was associated with increased BOLD response amplitudes in the left inferior occipital gyrus, right anterior parahippocampal gyrus, left triangular part of the inferior frontal gyrus, right middle temporal gyrus, left precuneus, left postcentral gyrus, right caudate, left supplementary motor cortex in HCs. For MCI, sadness was associated with increased BOLD response amplitudes in the right middle temporal gyrus, right inferior occipital gyrus, left middle frontal gyrus, and left superior parietal lobule. Figure 5 shows the activation patterns for the sadness condition, based on the contrast of sadness faces vs. neutral faces (sadness > neutral), for each group separately: left—HCs and right—patients with MCI.

**Fig. 5.**
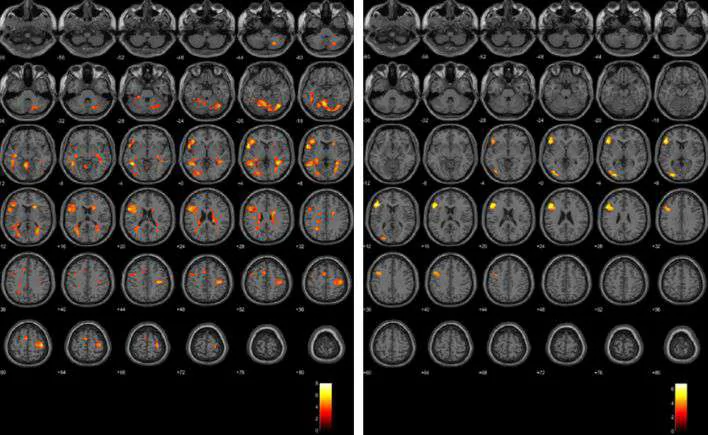
The activation patterns for the sadness condition, based on the contrast of sadness faces vs. neutral faces (sadness > neutral), for each group, are shown separately: left—HCs and right—patients with MCI [*p* < 0.05; FWE corrected at the cluster level].

**Fear** was associated with increased BOLD response amplitudes in the left inferior occipital, left triangular inferior frontal gyrus, right anterior parahippocampal, right caudate in HCs. Whereas fear had BOLD response amplitudes in the left inferior occipital gyrus and left triangular part of the inferior frontal gyrus, in the patients with MCI. Figure 6 shows the activation patterns for fear, based on the contrast of fear faces vs. neutral faces (fear > neutral), for each group separately: left—HCs and right—Patients with MCI.

**Fig. 6.**
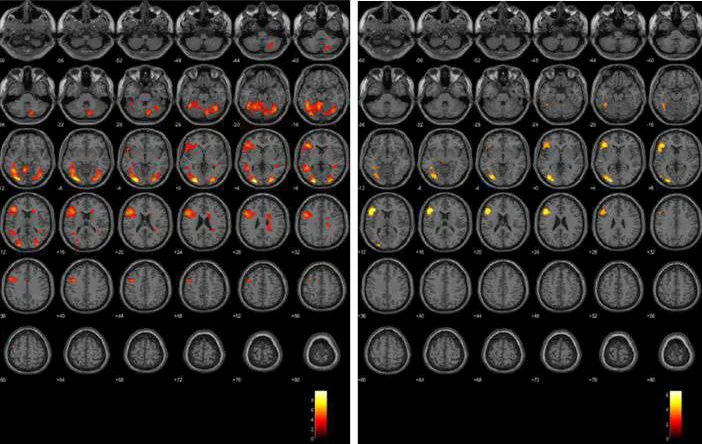
The activation patterns for the fear condition, based on the contrast of fear faces vs. neutral faces (fear > neutral), are shown for each group separately: left—HCs and right—patients with MCI [*p* < 0.05; FWE corrected at the cluster level].

For **happiness**, HCs showed significant activation in the left anterior cingulate gyrus and the temporal pole. Happiness did not elicit any significant activation in patients with MCI. Figure 7 shows the activation patterns for the happiness condition, based on the contrast of happiness faces vs. neutral faces (happiness > neutral), for each group separately: left—HCs and right—patients with MCI.

**Fig. 7.**
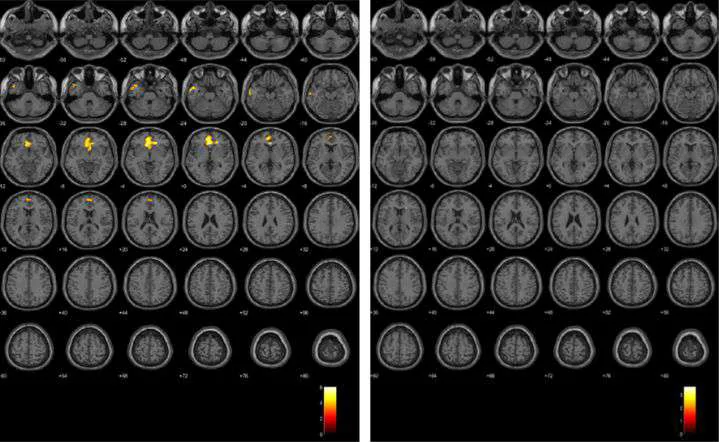
The activation patterns for the happiness condition based on the contrast of happiness faces vs. neutral faces (happiness > neutral), each group are shown separately: left—HCs and right—patients with MCI [*p* < 0.05; FWE corrected at the cluster level].

Next, we conducted a between-group comparison in which we compared BOLD response amplitudes for the four emotions between HCs and MCIs separately. This group-level analysis yielded no significant differences in BOLD responses amplitude for any emotion-specific contrast (HC>MCI or MCI > HC). Mean framewise displacement and total intracranial volume were additionally included as covariates across all between-group comparisons, and results remained robust to these controls. Of the regions hypothesized in H3, the fusiform gyrus was observed for anger versus neutral in HCs, and fronto-occipital regions were observed for fear versus neutral in HCs. However, the amygdala and several other hypothesized regions did not reach significance in either group.

## 4 Discussion

This study aimed to determine whether patients with MCI exhibit reduced facial emotion recognition performance compared with HCs, and whether any group differences are related to cognitive or socio-emotional characteristics. We also expected that patients with MCI would show reduced BOLD response amplitudes in core emotion-processing regions such as the fusiform gyrus, amygdala, posterior superior temporal sulcus, anterior temporal cortex, insula, secondary somatosensory cortex, ventrolateral prefrontal cortex, and dorsomedial prefrontal cortex, and that this reduction would be particularly pronounced for negative emotions (anger, fear, sadness). Our findings supported the predicted sadness-specific behavioral impairment, but did not support the expected group differences in overall emotion recognition or BOLD response amplitudes. Although overall emotion recognition performance did not differ between groups, patients with MCI showed a selective impairment in recognizing sadness, while recognition of happiness remained intact. Neither face processing ability (as measured by the facial composite task), emotional interference (i.e., the emotion Stroop effect reaction times as measured by the emotion Stroop task), nor performance across cognitive domains accounted for this deficit. Finally, the fMRI analyses revealed no significant group-level differences in BOLD response amplitudes for overall emotion processing or for specific emotional expressions.

### 4.1 Sadness recognition deficits and cognitive factors in MCI

In our first hypothesis, we expected that patients with MCI would perform worse in facial emotion recognition than HCs. We also examined whether demographic variables (such as age, gender, and education), face processing, EI, and performance across cognitive domains (attention, memory, language, executive functioning, and visuospatial skills) were associated with facial emotion recognition performance, and whether these factors could account for group differences. H1a was not supported in terms of overall facial emotion recognition performance, as no significant group difference was observed. However, emotion-specific analyses revealed significantly lower accuracy for sadness recognition in patients with MCI. Although patients with MCI showed numerically lower accuracy across all emotions, including happiness, the group difference reached statistical significance only for sadness. Effects for anger and fear were smaller and did not reach statistical significance. This pattern aligns with prior findings suggesting that patients with MCI show more pronounced difficulties in recognizing negative emotions (Elferink et al., 2015; García-Casal et al., 2019; McCade et al., 2013b; Richard-Mornas et al., 2012; Sarabia-Cobo et al., 2015). Older adults have also been shown to exhibit a “positivity bias”, characterized by relatively preserved processing of positive emotions but impaired recognition of negative emotions (Di Domenico et al., 2015). Consistent with this view, Wolpe et al. (2025) found that older adults typically require more visual information to identify negative facial expressions. However, they can recognize positive expressions with comparatively less information, even after accounting for general face-recognition ability (Wolpe et al., 2025).

Early structural or functional changes in key regions such as the amygdala and hippocampus, as well as changes in their connectivity with visual processing areas, can weaken the relationship between perception and emotional knowledge (Zheng et al., 2017). This makes it more difficult for patients with MCI to interpret subtle emotional cues. Negative emotion impairments may also arise because their expressions are often subtle or variable. A smile, for example, is a highly consistent and visually distinctive cue for happiness (Calvo & Marrero, 2009; Joshanloo & Weijers, 2025), whereas other negative expressions rely on more nuanced and low-intensity signals. For example, sadness is typically conveyed through more nuanced signals such as downward gaze, raised inner eyebrows, lip-corner depression, reduced walking speed, and slumped posture (Arias et al., 2020). These features may make sadness inherently more difficult to identify. Sadness also relies heavily on low-arousal cues that overlap with those of other emotions, increasing perceptual ambiguity. Calvo & Nummenmaa (2016) reported that sadness is often mistaken for neutral expression (12–17%) or other low-arousal negative emotions, such as disgust (15%) and fear (16%). This suggests that the cues for sadness are not unique and frequently overlap with other emotions that have similar cues (Calvo & Nummenmaa, 2016). Because negative expressions tend to be more ambiguous than positive ones, accurate recognition may depend more strongly on fine-grained visual analysis and the retrieval of stored emotional templates. In MCI, where perceptual integration or predictive processing mechanisms may already be weakened, these subtle cues may provide insufficient information for reliable interpretation, making sadness particularly vulnerable to misclassification.

Given that neutral expressions can be perceptually similar to sadness, we further examined whether the sadness-specific group difference could primarily reflect confusion with neutral faces. Recognition accuracy for neutral expressions did not differ significantly between patients with MCI and HCs (follow-up univariate ANOVA, *p* = 0.810). Moreover, supplementary trial-level analyses showed that sadness recognition did not improve when neutral was removed as a response option in either group; instead, errors were redistributed mainly toward fear and anger. These findings suggest that the reduced sadness recognition in MCI was not primarily driven by confusion between sadness and neutral expressions at the behavioral level (see Supplementary Table S14). Although sadness recognition is often described as relying on subtle configural facial processing (Calvo & Nummenmaa, 2016; Richler & Gauthier, 2014), our findings did not support a direct relationship between face-processing ability and sadness recognition. Facial composite task performance was not associated with emotion recognition accuracy and did not explain the differences observed between groups. This suggests that the sadness-specific impairment observed in the present sample cannot be attributed solely to reduced face-perception ability.

Sadness recognition also depends on socio-emotional processes that are particularly susceptible to decline in MCI. Lee and Kim (2024) demonstrated that individuals who more readily identified sad expressions also showed greater attentional engagement with sad faces and increased empathic concern for distressed others (Lee & Kim, 2024). This suggests that sadness perception is closely linked to emotional resonance and the ability to orient attention toward another person’s distress. At the same time, empathic abilities appear to decline already during the MCI stage. Giacomucci et al. (2022) reported reduced cognitive empathy in patients with MCI. Because sadness recognition may rely more strongly on empathic concern and perspective taking than threat-based emotions such as anger or fear, early disruptions in empathy could disproportionately impair the recognition of sadness.

Difficulties in recognizing sadness have important interpersonal consequences. Misinterpreting subtle emotional cues can impair social communication, reduce empathy, and contribute to misunderstandings during everyday interactions. Over time, these difficulties may strain personal relationships and complicate caregiving situations, thereby increasing caregiver burden (McCade et al., 2013a). Such socio-emotional difficulties may ultimately contribute to social withdrawal and reduced quality of life in patients with MCI (Yu et al., 2022).

EI was likewise not associated with emotion recognition performance. One explanation is that EI is multi-componential; in our study, the TEIQUE assessed well-being, self-control, emotionality, and sociability, whereas previous research suggests that only specific components, such as emotionality, may relate directly to emotion-recognition ability (Davis et al., 2021). Similarly, emotional interference showed no association with recognition accuracy. Although emotional distractors can impair response inhibition in some contexts (Meléndez et al., 2020), our findings suggest that such interference does not meaningfully disrupt basic facial emotion recognition in MCI. Together, these results indicate that the observed sadness-specific deficit in MCI is unlikely to stem from face-processing problems, emotional self-awareness, or interference from emotional distractors, but may instead reflect broader perceptual and socio-emotional challenges involved in interpreting ambiguous negative emotions.

### 4.2 Neural activity during facial emotion recognition in MCI

In our second hypothesis, we expected that patients with MCI would show reduced overall processing of emotional faces compared with HCs in regions such as the fusiform gyrus, amygdala, posterior superior temporal sulcus, anterior temporal cortex, insula, secondary somatosensory cortex, ventrolateral prefrontal cortex, and dorsomedial prefrontal cortex. Moreover, our third hypothesis suggested that this reduction would be specific for the emotions anger vs neutral (amygdala, fusiform gyrus, superior temporal gyrus, right inferior frontal gyrus), fear vs neutral (amygdala, pulvinar, and the fronto-occipital regions), and sadness vs neutral (right orbitofrontal and ventromedial prefrontal cortex including the cingulate cortex). Several of the regions hypothesized prior to data collection showed activation in HCs during emotion processing, providing partial support for the expected neural pattern. During overall emotion processing, HCs showed activation in the inferior frontal gyrus, overlapping with the hypothesized ventrolateral prefrontal cortex. For anger versus neutral, activation was observed in the fusiform gyrus, consistent with H3. For fear versus neutral, fronto-occipital activation was also observed in HCs, again partially consistent with H3. However, these activations were not significantly greater in HCs than in patients with MCI. Other hypothesized regions, including the amygdala, posterior superior temporal sulcus, anterior temporal cortex, insula, and dorsomedial prefrontal cortex, did not show significant activation in either group. The absence of amygdala activation is notable given its established role in emotion processing. One possible explanation is that static facial stimuli presented at full intensity may engage amygdala responses less consistently than dynamic or ambiguous emotional expressions (Becker et al., 2012). Thus, although the present findings did not fully confirm the hypothesized activation pattern, the partial overlap between observed and hypothesized regions in HCs suggests that key components of the expected emotion-processing network were engaged during the task.

For overall emotion recognition, HCs exhibited widespread activation for emotional faces relative to neutral, reflecting greater BOLD response amplitudes across regions commonly implicated in emotion recognition, including the inferior frontal gyrus (Tabei, 2015), right caudate (Graff-Radford et al., 2017), cerebral vermis I–IV (Clausi et al., 2017; Turner et al., 2007), and posterior cingulate gyrus (M. L. Phillips et al., 1998). In contrast, patients with MCI showed more restricted patterns of activation, involving the frontal and occipital regions. However, the difference in the spatial extent of neural engagement was not significant in group-level comparisons of BOLD response amplitudes. While examining individual emotions, HCs demonstrated wide and differentiated activation patterns for anger vs neutral (left opercular part of the inferior frontal gyrus, left fusiform gyrus, right occipital fusiform gyrus, right postcentral gyrus, left central operculum, and left supplementary motor cortex), fear vs neutral (left inferior occipital gyrus, left triangular part of the inferior frontal gyrus, right anterior parahippocampal gyrus, right caudate), sadness vs neutral (left inferior occipital gyrus, right anterior parahippocampal gyrus, left triangular part of the inferior frontal gyrus, right middle temporal gyrus, left precuneus, left postcentral gyrus, right caudate, and the left supplementary motor cortex), and happiness vs neutral (left anterior cingulate gyrus and the left temporal pole). Whereas patients with MCI exhibited more restricted activation patterns for anger vs neutral (left triangular part of the inferior frontal gyrus and left Inferior occipital gyrus), fear vs neutral (left inferior occipital gyrus and left triangular part of the inferior frontal gyrus), and sadness vs neutral (right middle temporal gyrus, right inferior occipital gyrus, left middle frontal gyrus, and left superior parietal lobule). Patients with MCI did not show significant activation in response to happiness vs neutral. Yet, similar to overall recognition, group-level comparisons for specific emotions showed no significant differences. Post-hoc power analyses indicated that the current sample of 30 participants per group was well powered to detect large between-group differences in BOLD response amplitudes (*d* = 0.80; power = 0.92), and moderately powered for medium effects (*d* = 0.50; power = 0.61). Accordingly, given the current sample size, there was adequate power to detect large between-group effects, while smaller differences in neural activity cannot be ruled out.

One possible explanation for the absence of statistically significant group differences is that neural alterations during emotion processing may be subtle at this stage of cognitive decline and, therefore, difficult to detect with the current sample size. Because MCI encompasses individuals who progress, remains stable, or even reverts to unimpaired cognition (Hu et al., 2017), it is likely that many participants in our sample were in a mild or early-phase stage of impairment, where neural alterations are subtle and fall below the detection threshold of conventional fMRI analyses. Another reason could be that the patients with MCI may rely on alternative neural pathways that are potentially less efficient, or that act as a form of compensation (Kanishka & Jha, 2022; Navas et al., 2015). These pathways do not engage traditional emotional processing circuits to the same extent, and this process has been termed “compensatory scaffolding” (Sherman et al., 2017). Such compensatory processes have been documented across cognitive domains and may enable patients to perform tasks despite diminished specialization in regions typically associated with emotion processing. For example, a study by Deschwanden et al. (2025) demonstrated that healthy older adults exhibited functional connectivity-based compensation, whereby increased connectivity in specific networks is associated with reduced cognitive decline despite structural deterioration, and the degree of compensation is influenced by several factors, including demographic, biological, and lifestyle (Deschwanden et al., 2025). If such compensatory processes are occurring, they may involve greater engagement of alternative brain regions in patients with MCI relative to HCs. Consistent with this interpretation, our complementary structural MRI paper found that greater left superior parietal lobule volume was associated with better sadness recognition in patients with MCI but not in HCs, suggesting that this region may serve as a structural reserve during early neurodegeneration (Mahadevan et al., 2026b). Notably, the left superior parietal lobule also appeared in the MCI sadness activation pattern in the present study, supporting the idea that parietal involvement may reflect compensatory processing during emotion recognition in MCI.

Alternatively, variability in the amount of structural brain loss among individuals might mean that some patients are relatively unaffected while others are more affected, such that group-level differences would only be detectable in very large samples. Importantly, the neural mechanisms underlying task performance may vary considerably across patients with MCI, such that similar behavioral impairments may emerge from different patterns of neural alteration or brain reserve, thereby reducing detectable group-level consistency in BOLD responses (Stern et al., 2020). Beyond severity, heterogeneity in cognitive deficit profiles may also have contributed to the absence of significant group differences. Patients with amnestic and non-amnestic MCI may process emotional information differently (McCade et al., 2013b), and this variability may have increased dispersion in BOLD responses, thereby reducing sensitivity to detect consistent between-group effects. These findings are consistent with our behavioral results indicating preserved recognition of positive emotions. This suggests that early socio-emotional decline is selective rather than global. Together, these considerations emphasize the complexity of interpreting fMRI data in aging and clinical populations and highlight the importance of integrating behavioral and neural measures to gain a comprehensive view of socio‑emotional functioning in MCI (Shi et al., 2025).

### 4.3 Task design considerations

Another important consideration is the type and complexity of the facial emotion recognition task used in the scanner. Initially, we piloted a version that used dynamic video stimuli with gradually increasing emotional intensity (from neutral expressions to full intensity, 0% to 100%). Nevertheless, this method did not result in reliable neural activity in the anticipated emotion-processing regions, likely due to the continuous change in intensity that made it challenging to identify the precise moment of peak emotional expression for analysis. Therefore, to ensure experimental control and comparability across participants, we selected static facial images for the final task. In addition, our task required responses using multiple buttons to capture nuanced answers. Such response demands can be challenging for patients with MCI due to slower reaction times or increased decision-making effort. However, overall performance indicated that participants could reliably follow the instructions.

### 4.4 Strengths and Limitations

Our study has several notable strengths, including its multimodal design that combines behavioral testing with task-based fMRI to investigate the relationship between emotion recognition and neural function in MCI. By focusing on both overall and emotion-specific functions, we were able to capture a detailed picture of emotional functioning. Furthermore, we investigated emotion recognition not as an isolated function but in relation to several cognitive domains. Interestingly, we observed behavioral impairments in emotion recognition without corresponding robust group-level neural differences, highlighting the potential value of behavioral measures for identifying subtle socio-emotional changes in a clinical setting.

However, some limitations should be acknowledged. An a priori power analysis was conducted prior to data collection using GPower with linear multiple regression (f² = 0.35, α = 0.05, power = 0.95), indicating a minimum required sample of 74 participants. However, due to recruitment constraints and the lack of available non-amnestic MCI participants, we had to conduct a post-hoc analysis and conclude our analysis with 60 participants (this included both patients with amnestic MCI and non-amnestic MCI; however, there were more patients with aMCI). Another limiting factor was the lack of caregiver burden data, which was collected via the Zarit Burden Interview (ZBI), and which was intended to associate the patient’s emotion recognition abilities with the caregiver burden that their families are facing. This variable was not included in the final analysis, as the data showed minimal variability and no participant scored more than a few points on the scale. Future studies should consider using a broader tool to capture caregiver burden in the context of the patient’s family. Another consideration is the unequal number of negative (anger, sadness, fear) versus positive (happiness) conditions. Although the asymmetry in emotion categories could theoretically bias the detection of emotion-specific effects, several aspects of our findings argue against a purely frequency-driven explanation. First, sadness was the only emotion that remained significantly impaired, despite equal presentation frequency across all negative emotions. Second, happiness showed no group effect. This pattern suggests that the observed deficit was tied to a specific emotion rather than to general exposure to negative expressions. Nevertheless, future studies should incorporate a more balanced valence structure (e.g., multiple positive emotions) to more precisely evaluate emotion-specific effects in MCI.

### 4.5 Future directions

Our findings point toward several avenues for future research. First, our emotion recognition task involved static images of models displaying different facial emotions. However, future studies could consider using dynamic stimuli, such as videos incorporating audio and visual elements, or even avatars, to better mimic real-life situations and gain a deeper understanding of the audio-visual neural circuits involved in processing emotions in daily life. Incorporating eye-tracking measures would offer further insights into how participants visually scan facial features when interpreting emotions. From a clinical perspective, using the facial composite task highlighted the importance of feature-based processing. Building on this, targeted interventions could be developed to train patients with MCI to pay attention to different facial features using various methods, including ambulatory assessment methods that could be implemented as a smartphone application to help people navigate real-life situations. For instance, participants could be prompted to focus on the eyes, nose, and mouth of another person when interacting with them, to understand their emotions and the context of the situation.

## 5 Conclusion

This study provides clear evidence that emotion recognition in MCI is not globally impaired but follows a specific pattern. We showed that patients with MCI perform similarly to HCs for most emotions and that difficulties arise primarily for sadness, an expression that relies on subtle and low-intensity facial cues. By integrating detailed behavioral testing with fMRI, our study demonstrates that this selective sadness difficulty cannot be explained by cognitive performance, face processing, EI, or interference from emotional information. This highlights that emotion-specific changes can be identified even when broader socio-emotional abilities remain preserved. A key contribution of this work is the combination of behavioral and neural measures. Although patients with MCI showed more restricted within-group activation patterns, whole-brain between-group analyses did not reveal statistically significant differences in BOLD response amplitudes for overall emotion processing or for individual emotion contrasts. Given the current sample size, small neural group differences cannot be ruled out. Together, these findings show that behavioral differences in sadness recognition were observed even though corresponding group differences in whole-brain BOLD response amplitudes were not detected.

## Supplementary Material

Supplementary Material

## Data Availability

Due to ethical restrictions, the data and code supporting the findings of this study are not publicly available. They can be requested from the corresponding author.

## Pre-Registration

Our study was pre-registered prior to data collection at the Open Science Framework (https://osf.io/yn3gp) on the 17th of October, 2023. A preprint version of this manuscript was published on PsyArXiv Preprints on 2nd of September 2025, and is available at https://osf.io/preprints/psyarxiv/t7vks_v1.
