# Supplementary material for: Negative emotion recognition impairments despite preserved neural activity in patients with mild cognitive impairment (MCI)
Source: Imaging Neurosci (Camb). 2026 Nov 2;4:IMAG.a.1428. doi: 10.1162/IMAG.a.1428 (PMC13728934; doi:10.1162/IMAG.a.1428)
Supplement: Supplementary file 1 [file IMAG.a.1428_supp.pdf]

Supplementary information for:

Negative Emotion Recognition Impairments Despite Preserved Neural Activity in Patients with Mild Cognitive Impairment (MCI).

Authors

Rachana Mahadevan[1], Naomi Kristin Giesers[2], Thomas Liman[2,3,4], Karsten Witt[2,5], Andrea Hildebrandt[6], Mandy Roheger[1*], Stephanie Rosemann[7*]. *Shared last co-authorship

[1] Ambulatory Assessment in Psychology Lab, Department of Psychology, Carl von Ossietzky Universität Oldenburg

[2] University Clinic of Neurology, Evangelical Hospital Oldenburg, Oldenburg, Germany

[3] Charité – Universitätsmedizin Berlin, Center for Stroke Research Berlin, Charite Platz 1, 10117 Berlin, Germany

[4] German Center for Neurodegenerative Disease DZNE, Berlin, Germany

[5] Research Center Neurosensory Science, University of Oldenburg, Oldenburg, Germany

[6] Psychological Methods and Statistics, Department of Psychology, Carl von Ossietzky Universität Oldenburg

[7] Biological Psychology Lab, Department of Psychology, Carl von Ossietzky University Oldenburg

Corresponding author:

Rachana Mahadevan

Ambulatory Assessment in Psychology, Department of Psychology

Carl von Ossietzky Universität Oldenburg, Ammerländer Heerstraße 114-118, 26129 Oldenburg, Germany

Tel. +49 4417985141, Email: rachana.mahadevan@uni-oldenburg.de

Orcid ID: https://orcid.org/0009-0002-5191-6417

Tables:

Table S1. TREND Statement Checklist.

Paper Section/ Topic	Item No	Descriptor	✓
Title and Abstract	1	• Information on how unit were allocated to interventions	✓
		• Structured abstract recommended	✓
		• Information on target population or study sample	✓
Background	2	• Scientific background and explanation of rationale	✓
		• Theories used in designing behavioral interventions	✓
Participants	3	• Eligibility criteria for participants, including criteria at different levels in recruitment/sampling plan (e.g., cities, clinics, subjects)	✓
		• Method of recruitment (e.g., referral, self-selection), including the sampling method if a systematic sampling plan was implemented	✓
		• Recruitment setting	✓
		• Settings and locations where the data were collected	✓
Interventions	4	• Details of the interventions intended for each study condition and how and when they were actually administered, specifically including:	
		○ Content: what was given?	
		○ Delivery method: how was the content given?	
		○ Unit of delivery: how were the subjects grouped during delivery?	
		○ Deliverer: who delivered the intervention?	
		○ Setting: where was the intervention delivered?	
		○ Exposure quantity and duration: how many sessions or episodes or events were intended to be delivered? How long were they intended to last?	
		○ Time span: how long was it intended to take to deliver the intervention to each unit?	
		○ Activities to increase compliance or adherence (e.g., incentives)	
Objectives	5	• Specific objectives and hypotheses	✓
Outcomes	6	• Clearly defined primary and secondary outcome measures	✓
		• Methods used to collect data and any methods used to enhance the quality of measurements	✓
		• Information on validated instruments such as psychometric and biometric properties	
Sample Size	7	• How sample size was determined and, when applicable, explanation of any interim analyses and stopping rules	✓

Assignment Method	8	• Unit of assignment (the unit being assigned to study condition, e.g., individual, group, community)	✓
		• Method used to assign units to study conditions, including details of any restriction (e.g., blocking, stratification, minimization)	✓
		• Inclusion of aspects employed to help minimize potential bias induced due to non-randomization (e.g., matching)	✓

Blinding (masking)	9	• Whether or not participants, those administering the interventions, and those assessing the outcomes were blinded to study condition assignment; if so, statement regarding how the blinding was accomplished and how it was assessed.	
Unit of Analysis	10	• Description of the smallest unit that is being analyzed to assess intervention effects (e.g., individual, group, or community)	
		• If the unit of analysis differs from the unit of assignment, the analytical method used to account for this (e.g., adjusting the standard error estimates by the design effect or using multilevel analysis)	
Statistical Methods	11	• Statistical methods used to compare study groups for primary methods outcome(s), including complex methods of correlated data	✓
		• Statistical methods used for additional analyses, such as a subgroup analyses and adjusted analysis	✓
		• Methods for imputing missing data, if used	✓
		• Statistical software or programs used	✓
Participant flow	12	• Flow of participants through each stage of the study: enrollment, assignment, allocation, and intervention exposure, follow-up, analysis (a diagram is strongly recommended)	✓
		○ Enrollment: the numbers of participants screened for eligibility, found to be eligible or not eligible, declined to be enrolled, and enrolled in the study	✓
		○ Assignment: the numbers of participants assigned to a study Condition	✓
		○ Allocation and intervention exposure: the number of participants assigned to each study condition and the number of participants who received each intervention	✓
		○ Follow-up: the number of participants who completed the follow- up or did not complete the follow-up (i.e., lost to follow-up), by study condition	
		○ Analysis: the number of participants included in or excluded from the main analysis, by study condition	✓

		• Description of protocol deviations from study as planned, along with reasons	
Recruitment	13	• Dates defining the periods of recruitment and follow-up	✓
Baseline Data	14	• Baseline demographic and clinical characteristics of participants in each study condition	✓
		• Baseline characteristics for each study condition relevant to specific disease prevention research	
		• Baseline comparisons of those lost to follow-up and those retained, overall and by study condition	
		• Comparison between study population at baseline and target population of interest	✓
Baseline equivalence	15	• Data on study group equivalence at baseline and statistical methods used to control for baseline differences	✓

Numbers analyzed	16	• Number of participants (denominator) included in each analysis for each study condition, particularly when the denominators change for different outcomes; statement of the results in absolute numbers when feasible	
		• Indication of whether the analysis strategy was "intention to treat" or, if not, description of how non-compliers were treated in the analyses	
Outcomes and estimation	17	• For each primary and secondary outcome, a summary of results for each estimation study condition, and the estimated effect size and a confidence interval to indicate the precision	
		• Inclusion of null and negative findings	✓
		• Inclusion of results from testing pre-specified causal pathways through which the intervention was intended to operate, if any	
Ancillary analyses	18	• Summary of other analyses performed, including subgroup or restricted analyses, indicating which are pre-specified or exploratory	
Adverse events	19	• Summary of all important adverse events or unintended effects in each study condition (including summary measures, effect size estimates, and confidence intervals)	✓
Interpretation	20	• Interpretation of the results, taking into account study hypotheses, sources of potential bias, imprecision of measures, multiplicative analyses, and other limitations or weaknesses of the study	✓
		• Discussion of results taking into account the mechanism by which the intervention was intended to work (causal pathways) or alternative mechanisms or explanations	✓
		• Discussion of the success of and barriers to implementing the intervention, fidelity of implementation	✓
		• Discussion of research, programmatic, or policy implications	✓
Generalizability	21	• Generalizability (external validity) of the trial findings, taking into account the study population, the characteristics of the intervention, length of follow-up, incentives, compliance rates, specific sites/settings involved in the study, and other contextual issues	✓
Overall Evidence	22	• General interpretation of the results in the context of current evidence and current theory	✓

Table S2

Descriptive statistics of demographic and cognitive variables across groups.

Variable	All participants (*n*=60)		MCI (*n*= 30)			HC (*n* = 30)			HC vs MCI		
	M	*SD*	*M*	*SD*	*range*	*M*	*SD*	*range*	*t*-value	*p*-value	*d*
Age	66.80	8.66	70.50	8.09	55-86	63.10	7.55	52-84	-3.644	**<0.001**	-0.941
Education	15.60	2.57	15.10	2.85	11-20	16.20	2.11	12-20	1.727	0.090	0.446
MoCA	25.40	2.71	23.10	1.55	19-25	27.70	1.25	26-30	12.572	**<0.001**	3.246
EI	5.51	0.70	5.27	0.74	3.83-6.77	5.74	0.58	4.27-6.77	5.740	**<0.05**	0.707
FCT	25.60	5.25	23.60	5.36	13-33	27.80	4.19	19-36	3.221	**<0.05**	0.846
Immediate Memory	-0.22	1.38	-1.04	1.09	-3.15-1.45	0.59	1.15	-1.25-2.54	5.564	**<0.001**	1.437
Delayed Memory	-0.58	0.79	-0.76	0.85	-2.42-1.26	-0.41	0.67	-1.79-1.15	1.787	0.080	0.461
Word Recognition	-0.34	1.07	-0.56	1.17	-2.5-1.2	-0.11	0.91	-2.65-1.1	1.664	0.101	0.430
Visuospatial recall	0.23	1.11	-0.16	1.30	-2.32-1.76	0.62	0.67	-1.06-1.61	2.931	**<0.05**	0.757
Visuospatial Construction	-0.01	1.06	-0.31	1.23	-3.23-1.35	0.29	0.75	-2.39-1.01	2.259	**<0.05**	0.583
Verbal Fluency	-0.11	0.98	-0.15	1.00	-1.89-1.48	-0.08	0.96	-1.81-1.75	0.269	0.800	0.069
Boston Naming Test	0.35	0.80	0.40	0.86	-1.6-1.77	0.30	0.74	-1.56-1.5	-0.504	0.616	-0.130
Phonematic Fluency	0.26	0.98	0.30	1.00	-2.29-2.6	0.22	0.96	-2.1-2.28	-0.302	0.763	-0.078
TMT A	0.77	0.98	0.70	0.93	-0.91-2.63	0.84	1.00	-1.58-2.63	0.541	0.590	0.140
TMT B	0.64	0.99	0.75	0.88	-0.85-2.78	0.54	1.07	-2.2-2.77	-0.808	0.422	-0.209

Note: M= mean, *SD*= standard deviation, MoCA= Montreal cognitive assessment, EI= Emotional intelligence, FCT= Facial Composite Task, TMT = Trail Making Test. The mean and

standard deviation of the z-scores of the tests (intended to measure attention (TMTA), memory (Immediate memory, delayed memory, word recognition, visuospatial recall), executive functioning (TMTB), language (verbal fluency, Boston naming test and phonematic fluency), and visuospatial construction are displayed and the p values represent the group comparisons between MCI (mild cognitive impairment) and HC (Healthy controls). Statistically significant results are shown in bold (p<0.001 and p<0.05).

Table S3

Group differences in Facial emotion recognition performance.

**Parameter**	**B**	**CI**	***p***	**FDR *p***
(Intercept)	0.16	[-0.21, 0.54]	**< 0.001**	**<0.001**
Group	-0.33	[-0.88, 0.23]	0.242	0.242
Age	-0.19	[-0.47, 0.09]	0.176	0.242

Residual standard error: 24.09 on 57 degrees of freedom, Multiple R-squared: 0.09143, Adjusted R-squared: 0.05955, F-statistic: 2.868 on 2 and 57 DF, p-value: 0.06504

Note: β displays the standardized β values, Confidence Interval (CI) =95%, and statistically significant results are shown in bold (p<0.001 and p<0.05). The group variable compares the emotion recognition abilities of the patients with mild cognitive impairment (MCI) and healthy controls (HC).

Table S4

Emotion specific performance across groups.

Effect	Wilks	approx_F	df1	df2	*p*
Group	0.790	2.83	5	53	**0.05**
Age	0.905	1.11	5	53	0.370
Residuals					
Anger		3.63	1	1	0.062
Fear		2.98	1	1	0.090
Happiness		1.41	1	1	0.241
Neutral		0.06	1	1	0.810
Sadness		9.85	1	1	**0.05**

Note: Statistically significant results are shown in bold (p<0.001 and p<0.05). The group variable compares the emotion recognition abilities of the patients with mild cognitive impairment (MCI) and healthy controls (HC).

Table S5

Association of Face processing and Facial emotion recognition task performance.

Effect	Wilks	approx_F	df1	df2	*p*
Group	0.755	3.18	5	49	**0.05**
Facial composite task	0.921	0.84	5	49	0.528
Age	0.938	0.64	5	49	0.668
Group x Facial composite task	0.911	0.96	5	49	0.452
Residuals					
Anger		3.34	1	1	0.073
Fear		4.39	1	1	**0.05**
Happiness		2.04	1	1	0.159
Neutral		0.20	1	1	0.658
Sadness		13.10	1	1	**0.001**

Note: Statistically significant results are shown in bold (p<0.001 and p<0.05). The group variable compares the emotion recognition abilities of the patients with mild cognitive impairment (MCI) and healthy controls (HC). N=2 participants did not complete the Facial composite task, and their missing values were accounted for in R.

Table S6

Association of Emotional Interference (Emotional Stroop Effect reaction time; as measured by the emotion stroop task) and Facial emotion recognition task performance.

Effect	Wilks	approx_F	df1	df2	*p*
Group	0.773	2.88	5	49	**0.05**
Emotion Interefrence	0.990	0.10	5	49	0.991
Age	0.911	0.96	5	49	0.452
Group x emotion interefrence	0.971	0.29	5	49	0.917
Residuals					
Univariate: Response Anger		4.41	1	1	**0.05**
Univariate: Response Fear		3.09	1	1	0.084
Univariate: Response Happiness		1.95	1	1	0.168
Univariate: Response Neutral		0.10	1	1	0.756

Univariate: Response Sadness	10.12	1	1	**0.05**

Note: Statistically significant results are shown in bold (p<0.001 and p<0.05). The group variable compares the emotion recognition abilities of the patients with mild cognitive impairment (MCI) and healthy controls (HC). N=2 participants did not complete the emotion stroop task due to color blindness, and their missing values were accounted for in R.

Table S7

Association of Language and Facial emotion recognition task performance.

**Parameter**	**B**	**CI**	***P***	**FDR *p***
(Intercept)	0.14	[-0.24, 0.52]	**< 0.001**	**<0.001**
Verbal fluency	-0.16	[-0.60, 0.28]	0.471	0.676
Group	-0.30	[-0.87, 0.27]	0.206	0.525
Boston Naming Test	0.01	[-0.40, 0.41]	0.972	0.972
Phonematic fluency	0.14	[-0.31, 0.60]	0.525	0.676
Age	-0.27	[-0.6, 0.06]	0.109	0.492
Verbal fluency x Group	0.27	[-0.30, 0.84]	0.349	0.629
Boston naming test x Group	0.32	[-0.21, 0.85]	0.233	0.525
Phonematic fluency x Group	0.07	[-0.49, 0.64]	0.796	0.896

Random effects: Residual standard error: 24.78 on 51 degrees of freedom, Multiple R-squared: 0.1949, Adjusted R-squared: 0.06861, F-statistic: 1.543 on 8 and 51 DF, p-value: 0.1658.

Note: β displays the standardized β values, Confidence Interval (CI) =95%, and statistically significant results are shown in bold (p<0.001 and p<0.05). The Verbal Fluency, Boston Naming Test, and Phonemic Fluency subtests assess the language domain within the neuropsychological test battery. The group variable compares the emotion recognition abilities of the patients with mild cognitive impairment (MCI) and healthy controls (HC). The interaction between each language test and the groups was used to assess whether language abilities contributed to better emotion recognition.

Table S8

Association of Memory and Facial emotion recognition task performance.

Parameter	B	CI	*P*	*FDR p*
(Intercept)	0.13	[-0.32, 0.58]	**< 0.001**	**< 0.001**
Immediate Memory	-0.40	[-0.79, -0.02]	**< 0.05**	0.089
Group	0.00	[-0.65, 0.64]	0.295	0.361
Delayed Memory	0.14	[-0.24, 0.53]	0.459	0.505
Word Recognition	-0.35	[-0.73, 0.03]	0.073	0.100
Visuospatial Recall	0.81	[0.28, 1.33]	**< 0.05**	**<0.05**
Age	-0.25	[-0.51, 0]	**<0.05**	0.091
Immediate Memory x Group	0.60	[-0.03, 1.23]	0.061	0.095
Delayed Memory x Group	-0.05	[-0.57, 0.47]	0.850	0.850
Word recognition x Group	0.63	[0.13, 1.14]	**< 0.05**	**0.057**
Visuospatial Recall x Group	-0.62	[-1.21, -0.03]	**<0.05**	0.089

Random effects: Residual standard error: 21.6 on 49 degrees of freedom, Multiple R-squared: 0.4125, Adjusted R-squared: 0.2926, F-statistic: 3.441 on 10 and 49 DF, p-value: 0.001768.

Note: β displays the standardized β values, Confidence Interval (CI) =95%, and statistically significant results are shown in bold ($p<0.001$ and $p<0.05$). The Immediate memory, Delayed memory, Word recognition, and Visuospatial recall subtests assess the memory domain within the neuropsychological test battery. The group variable compares the emotion recognition abilities of the patients with mild cognitive impairment (MCI) and healthy controls (HC). The interaction between each memory test and the groups was used to assess whether memory abilities contributed to better emotion recognition.

Table S9

Association of Visuospatial construction and Facial recognition task.

**Parameter**	**B**	**CI**	**P**	**FDR p**
(Intercept)	0.10	[-0.27, 0.47]	**< 0.001**	**< 0.001**
Visuospatial Construction	0.24	[-0.23, 0.70]	0.309	0.468
Group	-0.13	[-0.66, 0.4]	0.626	0.626
Age	-0.18	[-0.44, 0.07]	0.161	0.403
Visuospatial Construction x Group	0.24	[-0.30, 0.79]	0.375	0.468

Random effects: Residual standard error: 22.85 on 55 degrees of freedom, Multiple R-squared: 0.2616, Adjusted R-squared: 0.2079, F-statistic: 4.872 on 4 and 55 DF, p-value: 0.001954

Note: β displays the standardized β values, Confidence Interval (CI) =95%, and statistically significant results are shown in bold (p<0.001 and p<0.05). Visuospatial construction tests assess the visuospatial construction abilities within the neuropsychological test battery. The group variable compares the emotion recognition abilities of the patients with mild cognitive impairment (MCI) and healthy controls (HC). The interaction between the visuospatial construction and the groups was used to assess whether this ability contributed to better emotion recognition.

Table S10

Association of attention and facial emotion recognition task performance.

**Parameter**	**β**	**CI**	***p***	**FDR *p***
(Intercept)	0.15	[-0.23, 0.53]	**< 0.001**	**<0.001**
TMT A	0.11	[-0.25, 0.47]	0.552	0.690
Group	-0.32	[-0.89, 0.25]	0.698	0.698
Age	-0.20	[-0.52, 0.11]	0.200	0.500
TMT A x Group	-0.23	[-0.75, 0.28]	0.372	0.619

Random effects: Residual standard error: 25.16 on 55 degrees of freedom, Multiple R-squared: 0.1047, Adjusted R-squared: 0.03955, F-statistic: 1.607 on 4 and 55 DF, p-value: 0.1854.

Note: β displays the standardized β values, Confidence Interval (CI) =95%, and statistically significant results are shown in bold (p<0.001 and p<0.05. TMT A refers to the Trail Making

Test A test assessing attention abilities within the neuropsychological test battery. The group variable compares the emotion recognition abilities of the patients with mild cognitive impairment (MCI) and healthy controls (HC). The interaction between attention and the groups was used to assess whether this ability contributed to better emotion recognition.

Table S11

Association of executive functioning and facial emotion recognition task performance.

**Parameter**	**β**	**CI**	***P***	**FDR *p***
(Intercept)	0.17	[-0.2, 0.54]	**< 0.001**	**< 0.001**
TMT B	0.04	[-0.29, 0.36]	0.827	0.827
Group	-0.37	[-0.93,0.18]	0.074	0.184
Age	-0.19	[-0.47, 0.09]	0.177	0.250
TMT B x Group	0.33	[-0.18, 0.85]	0.200	0.250

Random effects: Residual standard error: 24.58 on 55 degrees of freedom, Multiple R-squared: 0.1456, Adjusted R-squared: 0.08347, F-statistic: 2.343 on 4 and 55 DF, p-value: 0.06605.

Note: β displays the standardized β values, Confidence Interval (CI) =95%, and statistically significant results are shown in bold ($p<0.001$ and $p<0.05$). TMT B refers to the Trail Making Test B test, which assesses executive functioning abilities within the neuropsychological test battery. The group variable compares the emotion recognition abilities of the patients with mild cognitive impairment (MCI) and healthy controls (HC). The interaction between executive functioning and the groups was used to assess whether this ability contributed to better emotion recognition.

Table S12

Association of the EI and the Facial emotion recognition task performance.

**Parameter**	**β**	**CI**	***p***	**FDR *p***
(Intercept)	0.10	[-0.30, 0.50]	**<0.05**	0.203
EI	0.22	[-0.22, 0.66]	0.327	0.545
Group	-0.25	[-0.83, 0.33]	0.726	0.726
Age	-0.17	[-0.45, 0.12]	0.251	0.545

Parameter	β	CI	*p*	FDR *p*
EI x Group	-0.13	[-0.69, 0.43]	0.637	0.726

Residual standard error: 25.07 on 55 degrees of freedom, Multiple R-squared: 0.111, Adjusted R-squared: 0.0463, F-statistic: 1.716 on 4 and 55 DF, p-value: 0.1595.

Note: β displays the standardized values, Confidence Interval (CI) =95%, and statistically significant results are shown in bold ($p<0.001$ and $p<0.05$). EI = Emotional Intelligence, while well-being, self-control, emotionality, and sociability are its subdomains. The group variable compares the emotion recognition abilities of the patients with mild cognitive impairment (MCI) and healthy controls (HC). The interaction between emotional intelligence and the groups was used to assess whether the relationship between emotional intelligence and emotion recognition differed.

Table S13

Association of demographic variables and Facial emotion recognition task performance.

Parameter	β	CI	*P*	FDR *p*
(Intercept)	0.50	[-0.05, 1.05]	**< 0.05**	0.190
Age	-0.02	[-0.49, 0.45]	0.932	0.962
Group	-0.48	[-1.24, 0.29]	0.962	0.962
Education	-0.07	[-0.55, 0.41]	0.776	0.962
Gender	-0.61	[-1.43, 0.21]	0.141	0.564
Age x Group	-0.18	[-0.8, 0.45]	0.574	0.962
Group x Education	0.12	[-0.47, 0.71]	0.690	0.962
Group x Gender	0.27	[-0.86, 1.39]	0.636	0.962

Residual standard error: 25.14 on 52 degrees of freedom, Multiple R-squared: 0.1553, Adjusted R-squared: 0.04156, F-statistic: 1.366 on 7 and 52 DF, p-value: 0.2397.

Note: β displays the standardized β values, Confidence Interval (CI) =95%, and statistically significant results are shown in bold ($p<0.001$ and $p<0.05$). The group variable compares the emotion recognition abilities of the patients with mild cognitive impairment (MCI) and healthy controls (HC). The gender variable compares the emotion recognition abilities of different genders who participated in the study. The interaction between participant gender and

stimulus gender was used to assess whether different participant genders were influenced by different stimulus genders.

Table S14

Sadness recognition with and without neutral as a response option.

Group	Neutral included	Neutral not included	*t*(29)	*p*
**HC**	80.7%	80.0%	0.28	0.782
**MCI**	70.4%	71.6%	-0.35	0.727

Note. Accuracy refers to sadness-recognition trials. Paired-samples t-tests compared sadness recognition accuracy when neutral was included versus not included as a response option within each group. Removing neutral as a response option did not significantly change sadness-recognition accuracy in either group. Incorrect responses were redistributed mainly toward fear and anger.
